# Supplementary figures and images for: The use of non-model Drosophila species to study natural variation in TOR pathway signaling
Source: PLoS One. 2022 Sep 22;17(9):e0270436. doi: 10.1371/journal.pone.0270436 (PMC9499319; doi:10.1371/journal.pone.0270436)

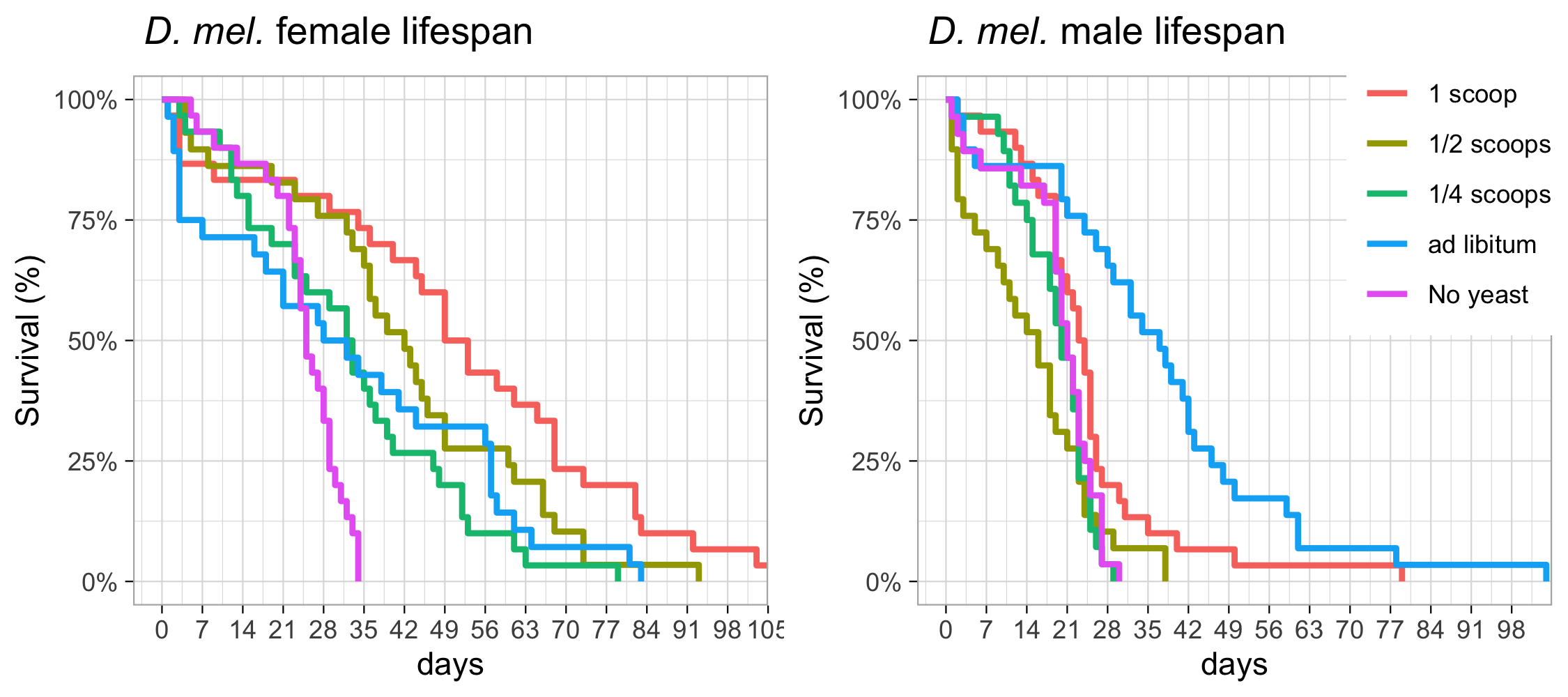

Supplement: S1 Fig — (TIF) [file pone.0270436.s001.tif]

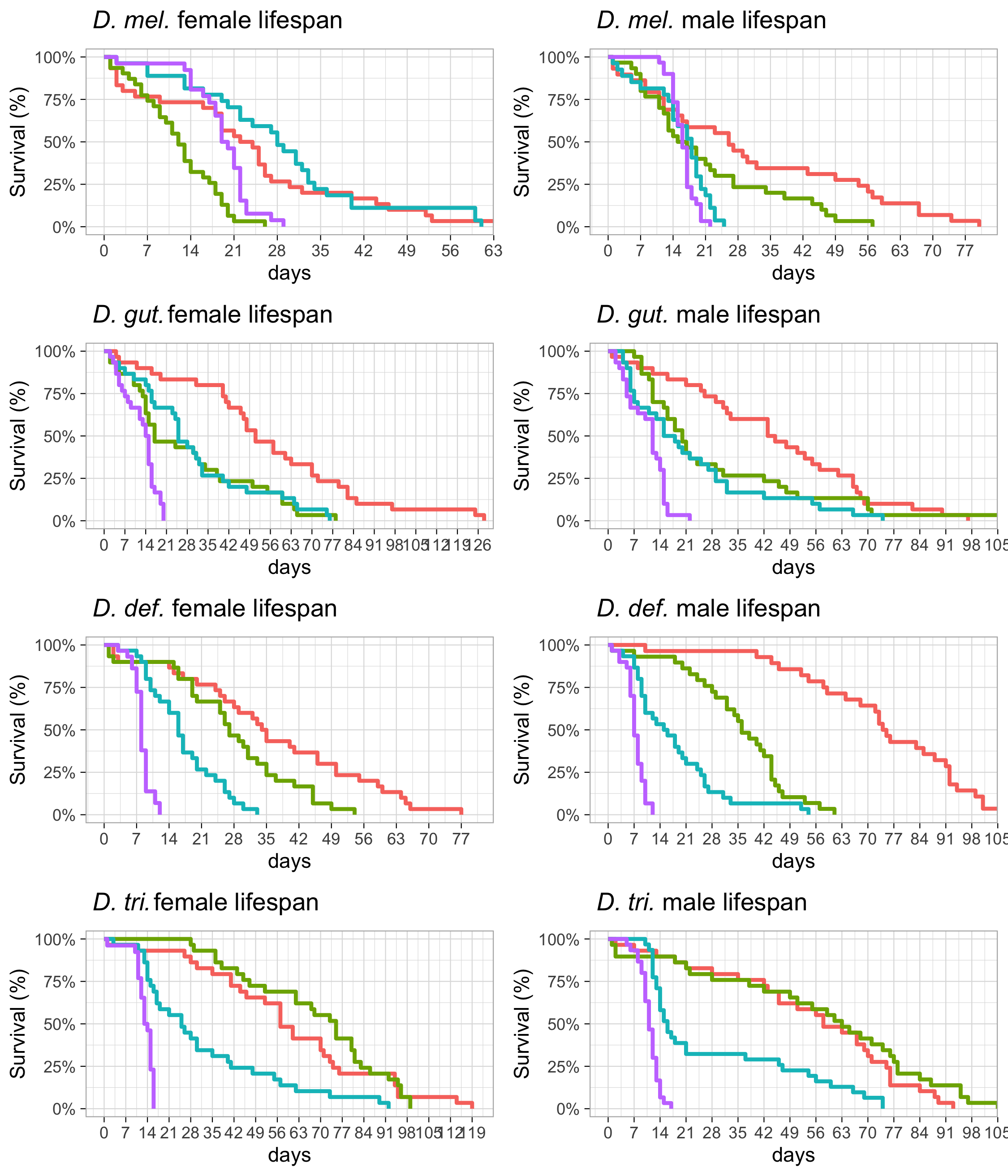

Supplement: S2 Fig — Survival curves for D. mel., D. gut., D. def., and D. tri. treated with diets ranging from no yeast (purple), minimum (1 scoop/fly/day, blue), to ad libitum (red), as well as an ad libitum yeast diet on rapamycin-treated medium (200 μM, green). Escaped or flies killed by accident were discarded. (TIF) [file pone.0270436.s002.tif]

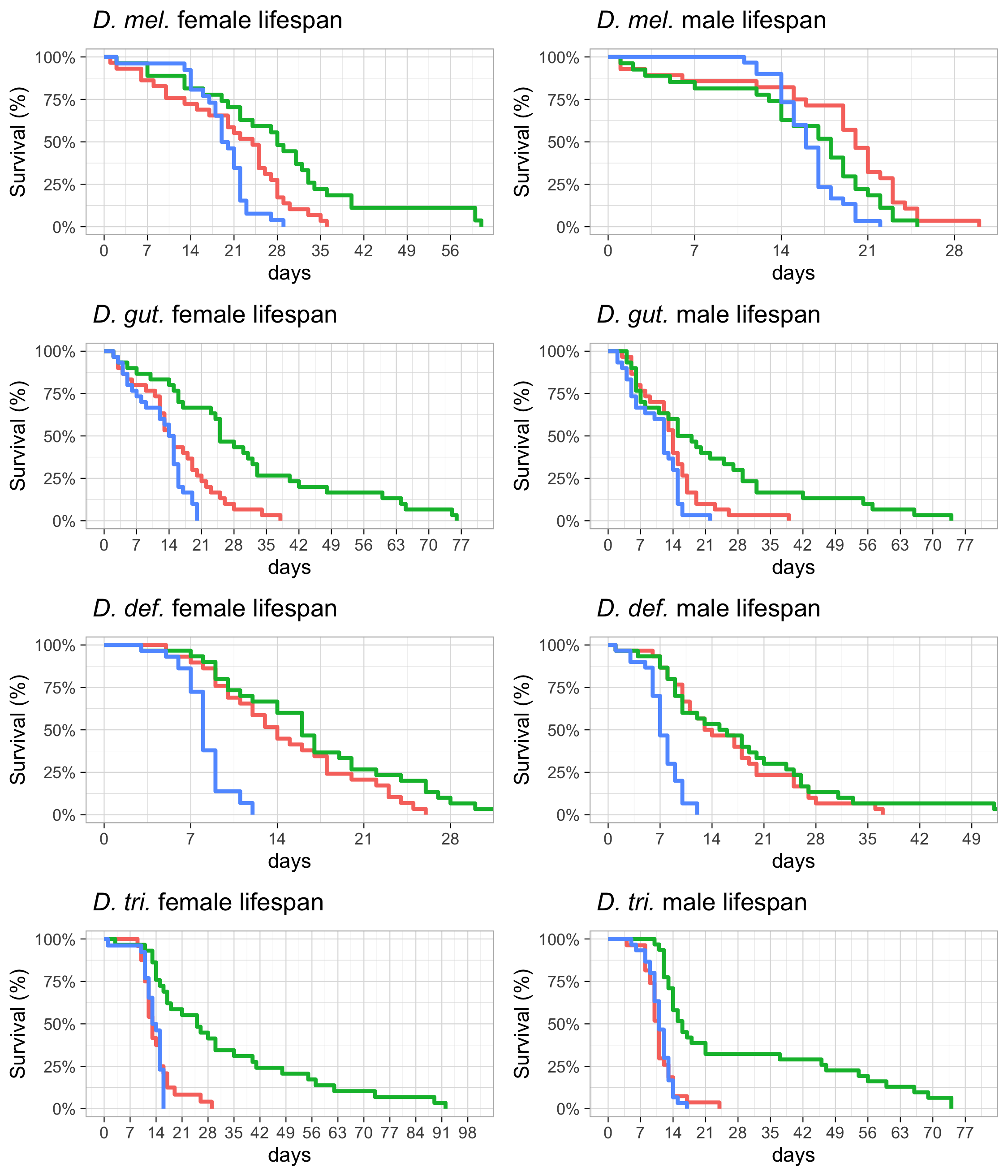

Supplement: S3 Fig — Female and male lifespan of D. mel., D. gut., D. def., and D. tri. treated with diets including no yeast (blue), minimum dead yeast (1 scoop/fly/day, red), and minimum alive yeast (1 scoop/fly/day, green). (TIF) [file pone.0270436.s003.tif]

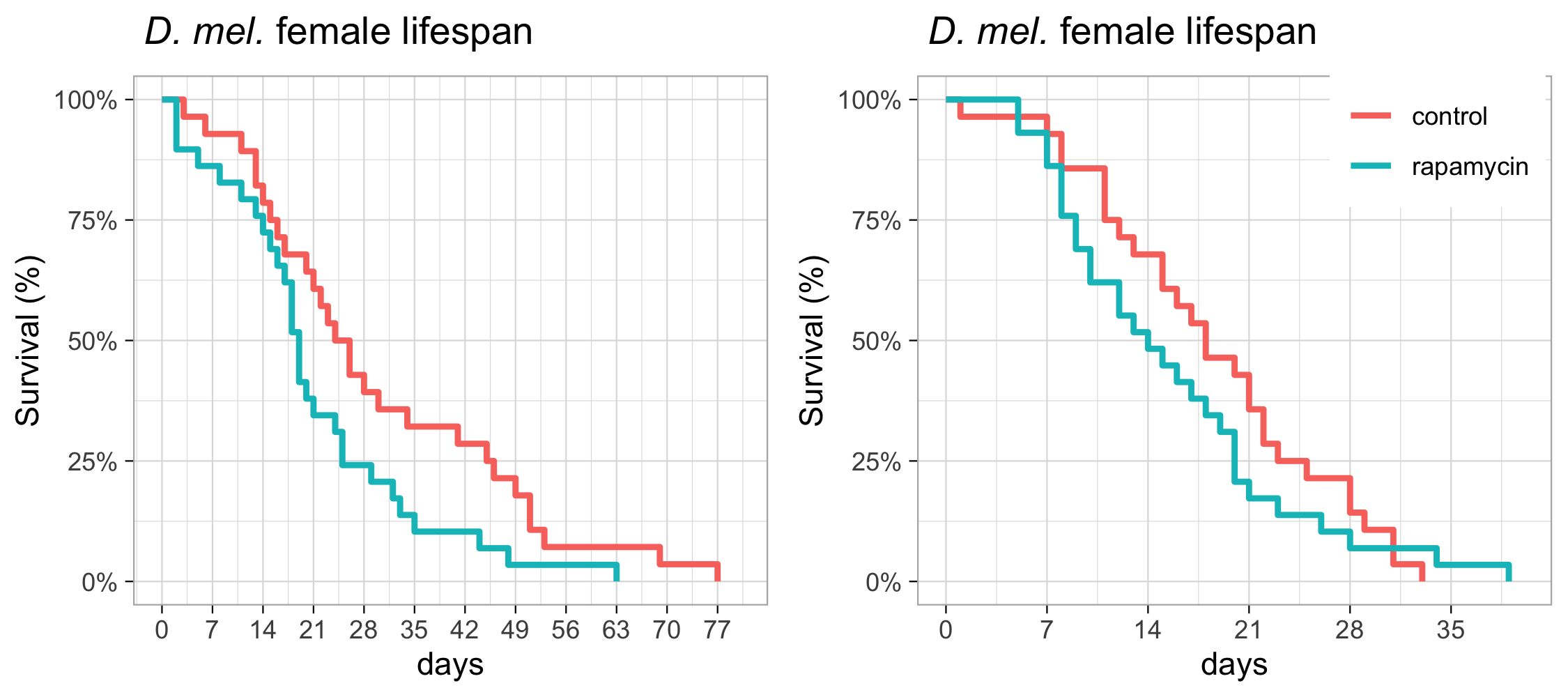

Supplement: S4 Fig — Survival curves for male and female lifespan in [days] for D. mel. on medium (2 scoops/fly/per day) diets with (blue) and without (red) rapamycin (200 μM). n = number of flies going into the analysis; escaped or flies killed by accident were discarded. (TIF) [file pone.0270436.s004.tif]
