# Supplementary material for: The use of non-model Drosophila species to study natural variation in TOR pathway signaling
Source: PLoS One. 2022 Sep 22;17(9):e0270436. doi: 10.1371/journal.pone.0270436 (PMC9499319; doi:10.1371/journal.pone.0270436)
Supplement: Attachment — Submitted filename: Response to reviewers.docx [file pone.0270436.s006.docx]

**Response to reviewers**

PONE-D-22-16667

The use of non-model Drosophila species to study natural variation in TOR pathway signaling

**Reviewer #1:**

This manuscript studies the fecundity and longevity of four different species of Drosophila. The main data shown by the authors is that as yeast concentration increases so does fecundity. The authors use an inhibitor of TOR pathway known to decrease fecundity to show that fecundity is inhibited when TOR pathway is inhibited. They also showed mean and median longevity studies.

**Comment:** However, little can be concluded from the longevity studies since no statistical power or survival analysis are provided. The data does not seem to support the claim about how non-model Drosophila species showed natural variation in TOR signaling.

**Response:** To address the lack of statistical analysis in the form of survival analysis and accompanying p-values, supplemental files were added showing all survival curves for the in-text tables. Additional p-values were included in in-text charts where appropriate and attached as a supplemental file. We also toned down the observation about the TOR regulation.

**Reviewer #2:**

In the manuscript, the authors examined the interspecific differences in nutrient signaling among four difference Drosophila species. Results showed that there may be natural variation in the TOR signaling based on Fecundity and longevity data. Results are interesting and there seems to be no major errors in experimental design and/or analysis. I have just minor suggestions.

**Comment 1:** Only 30 flies were tested in each group which will weaken the statistical analysis. Any reason not to increase the replicate number?

**Response 1:** The flies were split up into three replicates, each containing 10 males and 10 females. The flies were housed in small Petri dishes and anesthetized by hand to sort out any deaths and move them to the next day’s Petri dish. After moving the flies, the eggs were counted by hand. Due to the desire to have telling results while having manageable egg numbers, total female flies were limited to 30. Future projects could take advantage of automated counting.

**Comment 2:** More rigorous statistical analysis for longevity assay is necessary - there is no p values.

**Response 2:** To address the lack of statistical analysis in the form of survival analysis and accompanying p-values, five supplemental files (S1 – S4_Figs and S5_File) were added showing all survival curves for the in-text tables. Additional p-values were included in in-text charts where appropriate and attached as a supplemental file

**Comment 3:** Even though the authors compared the feeding rate among four Drosophila species, the differences in life-history data may be due to other factors like differences in microbiome and/or differences in digestive capacity. It should be discussed somewhere.

**Response 3:** To address the comment about the possible influence of the microbiota and/or digestive capacity, a paragraph was added to the limitations. In our long-term lab reared species, we believe that the microbiomes are similar, while more recent wild-caught flies likely resemble the natural state of the original region.

**Comment 4:** It seems that there were 10 males and 10 females in a single vial. Mating frequency is different between low and high yeast diets which will also affect longevity. The confounding effect of mating should be discussed also.

**Response 4:** We included a new section addressing these issues in the discussion as well as a paragraph in the limitations section. The discussion of the effect of increased mating on longevity is still ongoing and not entirely understood. It is, however, a factor to consider for further experimentation.
